# Metachronous multiple primary cancers involving pulmonary non-Hodgkin lymphoma and bladder cancer: a case report

**DOI:** 10.3389/fonc.2026.1734661

**Published:** 2026-02-25

**Authors:** Kunlei Tan, Xiyun Quan, Miduo Tan, Zhijun Han

**Affiliations:** 1 Department of Urology, Zhuzhou Central Hospital, Zhuzhou, Hunan, China; 2 Department of Pathology, Zhuzhou Central Hospital, Zhuzhou, Hunan, China; 3 Department of Breast Surgery, Zhuzhou Central Hospital, Zhuzhou, Hunan, China

**Keywords:** bladder cancer, multiple primary cancers, non-Hodgkin lymphoma, prevention, treatment

## Abstract

Multiple primary cancers (MPC) are characterized by the synchronous or metachronous occurrence of two or more distinct histopathological tumor types. We report a rare clinical case of a 65-year-old woman diagnosed with pulmonary mucosa-associated lymphoid tissue (MALT) marginal zone B-cell lymphoma and subsequent invasive bladder carcinoma. The patient, previously treated with multiple lines of chemotherapy for MALT lymphoma, was admitted to our hospital with lower abdominal and perineal pain accompanied by gross hematuria for one week. Cystoscopic examination and transurethral resection of the bladder lesion confirmed the diagnosis of bladder cancer. She later underwent laparoscopic radical cystectomy, and postoperative histopathological evaluation revealed invasive urothelial carcinoma infiltrating the cervical and vaginal fornix stroma.

## Introduction

Multiple primary cancers (MPC), also known as multiple primary malignancies, are defined as the occurrence of two or more histologically distinct primary malignant tumors in a single individual, arising either synchronously or metachronously in the same or different organs. Although relatively uncommon, MPC represents a clinically important and increasingly recognized phenomenon in modern oncology. The etiology of MPC is multifactorial and has been associated with improved cancer survival, long-term exposure to chemotherapy and/or radiotherapy, enhanced diagnostic sensitivity, as well as persistent genetic and behavioral risk factors.

With advances in oncologic research and therapeutic strategies, survival outcomes of patients with malignancies have markedly improved, resulting in a growing population of long-term cancer survivors. Consequently, the incidence of MPC has been steadily increasing. Epidemiological studies indicate that the most frequently reported MPC involve malignancies such as breast, liver, head and neck, and colorectal cancers. In contrast, metachronous MPC comprising non-Hodgkin lymphoma and bladder cancer remain exceedingly rare and are sparsely documented in the literature.

Herein, we report a rare case of metachronous non-Hodgkin lymphoma followed by invasive bladder carcinoma. This case aims to provide clinical insights into the diagnostic challenges, therapeutic decision-making, and potential pathogenetic mechanisms underlying this uncommon combination, thereby contributing to the growing body of literature on MPC in the era of prolonged cancer survival.

## Case presentation

**Figure 1:**
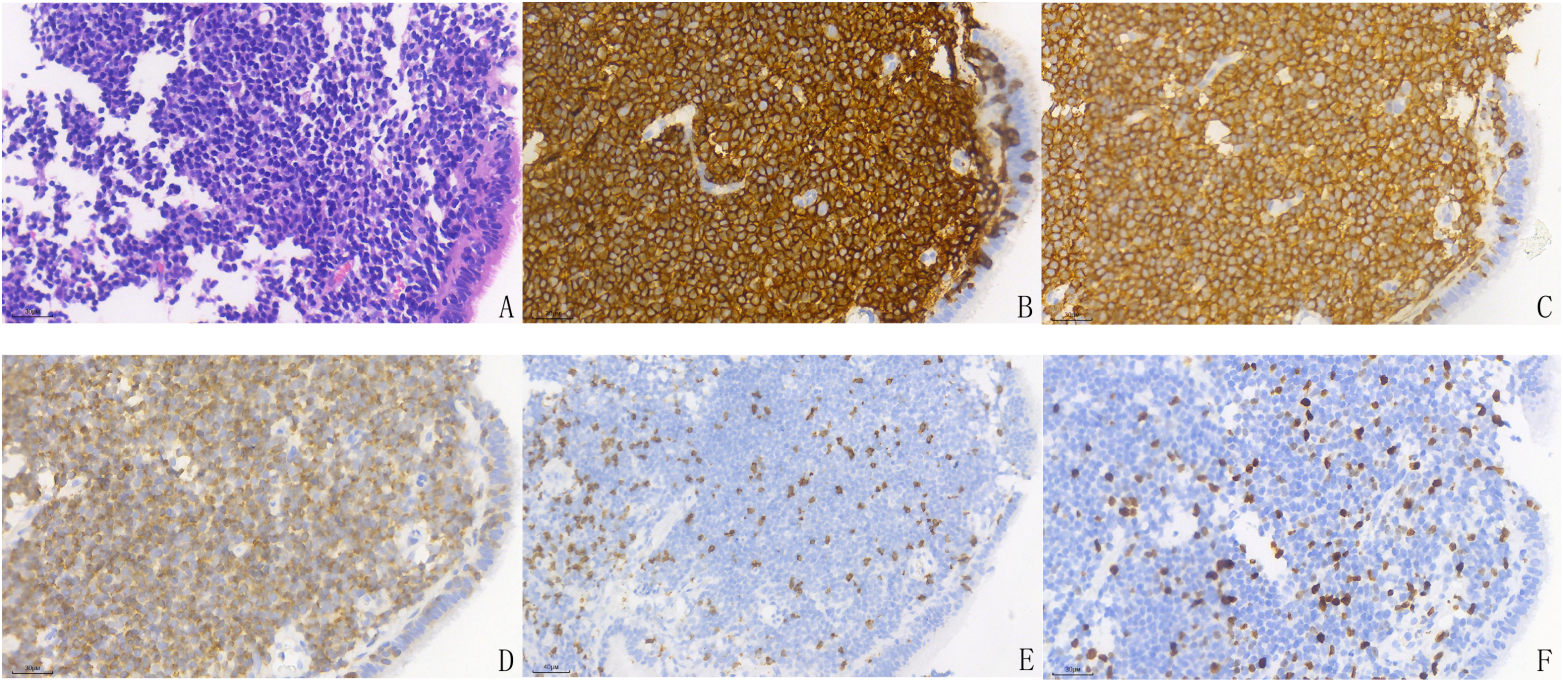
Pathological features of MALT components. HE staining **(A)** demonstrates a diffuse and patchy infiltration of densely packed small lymphoid cells, leading to architectural effacement of the MALT tissue. Immunohistochemical staining shows positive expression for CD20 **(B)**, CD79a **(C)**, BCL6 **(D)**, and CD3 **(E)**, with a Ki-67 proliferation index of approximately 10% **(F)**.

A 65-year-old female patient presented with a two-day history of lower abdominal and perineal pain accompanied by gross hematuria. The patient reported significant perineal distension and pain during urination, along with hematuria, urinary frequency, urgency, and dysuria, with no radiating pain. Abdominal examination revealed mild suprapubic tenderness without palpable masses or signs of peritoneal irritation. No costovertebral angle tenderness was noted. No superficial lymphadenopathy was detected in the cervical, axillary, or inguinal regions. Cardiopulmonary examination was unremarkable. Pelvic examination revealed no obvious abnormalities.A detailed review of her medical history revealed no family history of cancer and no major cancer risk factors. She denied a history of smoking or alcohol abuse and worked as a homemaker. Seven years prior to the current admission, the patient was hospitalized due to hoarseness and shortness of breath. A lung biopsy was performed, and histopathological examination of the left lung biopsy specimen revealed extensive lymphocyte proliferation. Immunohistochemical (IHC) analysis showed the following profile: CD3 (T-cell+), CD5 (T-cell+), CD20 (+++), CD79a (+++), Bcl-2 (+), Bcl-6 (focally positive in rare cells), CD21 (+), Ki-67 (approximately 10%+), CD10 (-), and Mum-1 (scattered positivity in rare cells) (**Figure 1**). The findings supported the diagnosis of non-Hodgkin MALT marginal zone B-cell lymphoma (left lung origin), stage IVA.The diagnosis of pulmonary MALT lymphoma was established based on characteristic histopathological features and an immunophenotypic profile consistent with extranodal marginal zone lymphoma. At the time of diagnosis, comprehensive clinical, radiological, and pathological evaluations revealed no evidence of other synchronous primary malignancies.

**Figure 2:**
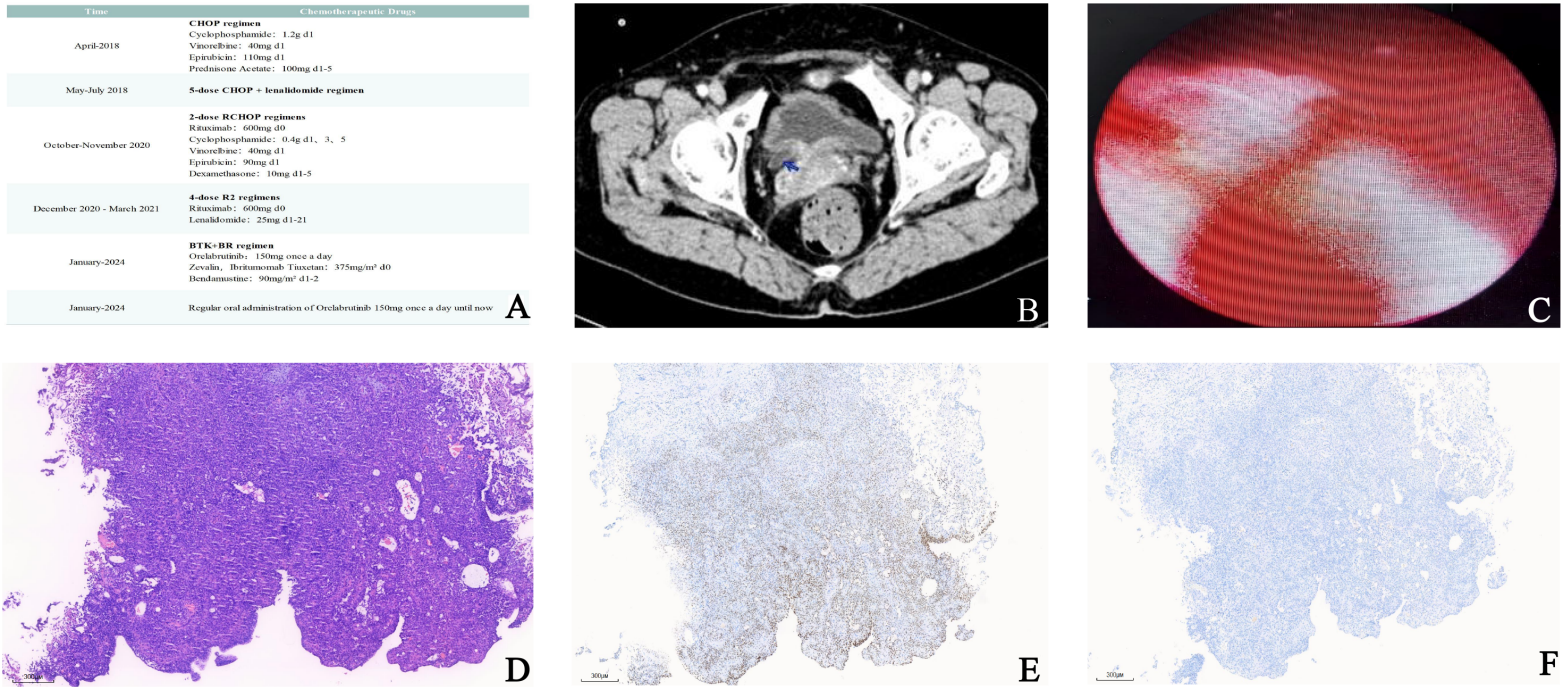
Previous chemotherapy history, radiological findings, and pathological results of bladder lesion resection. **(A)** Summary of the patient’s previous chemotherapy regimens. **(B)** Computed tomography (CT) revealed thickening and luminal narrowing of the intramural segment of the right ureter, along with diffuse thickening of the bladder wall. **(C)** Cystoscopic examination findings. **(D–F)** Histopathological examination results of bladder tumor: HE staining showed bladder urothelial carcinoma **(D)**. GATA3 and CK20 expression were negative. **(E, F)**.

Over the past seven years, the patient has received multiple chemotherapy regimens (**Figure 2A**). Following confirmation of the diagnosis, the patient initiated first-line therapy with the CHOP regimen (cyclophosphamide[CTX] 1.2 g on day 1, vinorelbine 40 mg on day 1, epirubicin 110 mg on day 1, and prednisone acetate 100 mg on days 1–5) in April 2016. Beginning in May 2016, she subsequently received five cycles of CHOP in combination with lenalidomide. However, after completing five cycles, the patient declined further treatment and remained without therapy or regular follow-up for nearly two years after discharge.

In October 2018, the patient was re-admitted because of dyspnea. Laboratory examinations, including a complete blood count, routine biochemistry, and coagulation profile, revealed no significant abnormalities. Computed tomography (CT) demonstrated that the lesions in the lingular segment of the left lung and the anterior basal segment of the left lower lobe were unchanged compared with previous imaging. Consequently, she initiated salvage therapy with two cycles of the R-CHOP regimen in October 2018 (rituximab 600 mg on day 0; CTX 0.4 g on days 1, 3, and 5; vinorelbine 40 mg on day 1; epirubicin 90 mg on day 1; and dexamethasone 10 mg on days 1–5).

In December 2018, given that the cumulative anthracycline dose was approaching the recommended safety threshold, the treatment strategy was switched to the R² regimen (rituximab 600 mg on day 0 and lenalidomide 25 mg on days 1–21), and the patient completed four cycles. The patient was clinically stable and declined further treatment.

The patient remained asymptomatic for more than two years. However, in January 2022, she was re-admitted with progressively worsening dyspnea that had persisted for six months, accompanied by night sweats. Laboratory examinations revealed the following results: white blood cell count, 7.35 × 10^9^/L; lymphocyte percentage, 12.9%; monocyte count, 1.92 × 10^9^/L; and monocyte percentage, 21.8%. CT imaging demonstrated further regression of the lesions in both the lingular segment and the anteromedial basal segment of the left lung compared with prior scans. The patient subsequently initiated therapy with a Bruton’s tyrosine kinase (BTK) inhibitor combined with the BR regimen (oral orelabrutinib 150 mg once daily, zebrutumab 375 mg/m² on day 0, and bendamustine 90 mg/m² on days 1–2). After discharge, she continued maintenance therapy with daily oral orelabrutinib (150 mg once daily).

Current urological CT imaging demonstrated thickening and narrowing of the right intramural ureter along with diffuse bladder wall thickening (**Figure 2B**). Cystoscopic examination revealed a prominent raised lesion near the right ureteral orifice (**Figure 2C**). Comprehensive evaluation identified clear surgical indications without contraindications. Intraoperative observation identified multiple cauliflower-like masses on the right bladder wall, measuring approximately 30 mm in diameter. The patient subsequently underwent transurethral resection of the bladder lesion. Postoperative pathological analysis confirmed the diagnosis of invasive bladder cancer (**Figures 2D–F**).

One month later, the patient underwent a follow-up surgical evaluation and subsequently received a radical cystectomy. The procedure was performed laparoscopically and included radical resection of the bladder, construction of an ileal conduit, and bilateral ureteral implantation. Notably, postoperative pathological examination of the adherent area involving the bladder and uterus revealed malignant tissue consistent with poorly differentiated carcinoma. The tumor was found to be infiltrating the full thickness of the bladder wall and involving the cervical and vaginal fornix stroma. No definite lymphovascular invasion or perineural involvement was identified.

**Figure 3:**
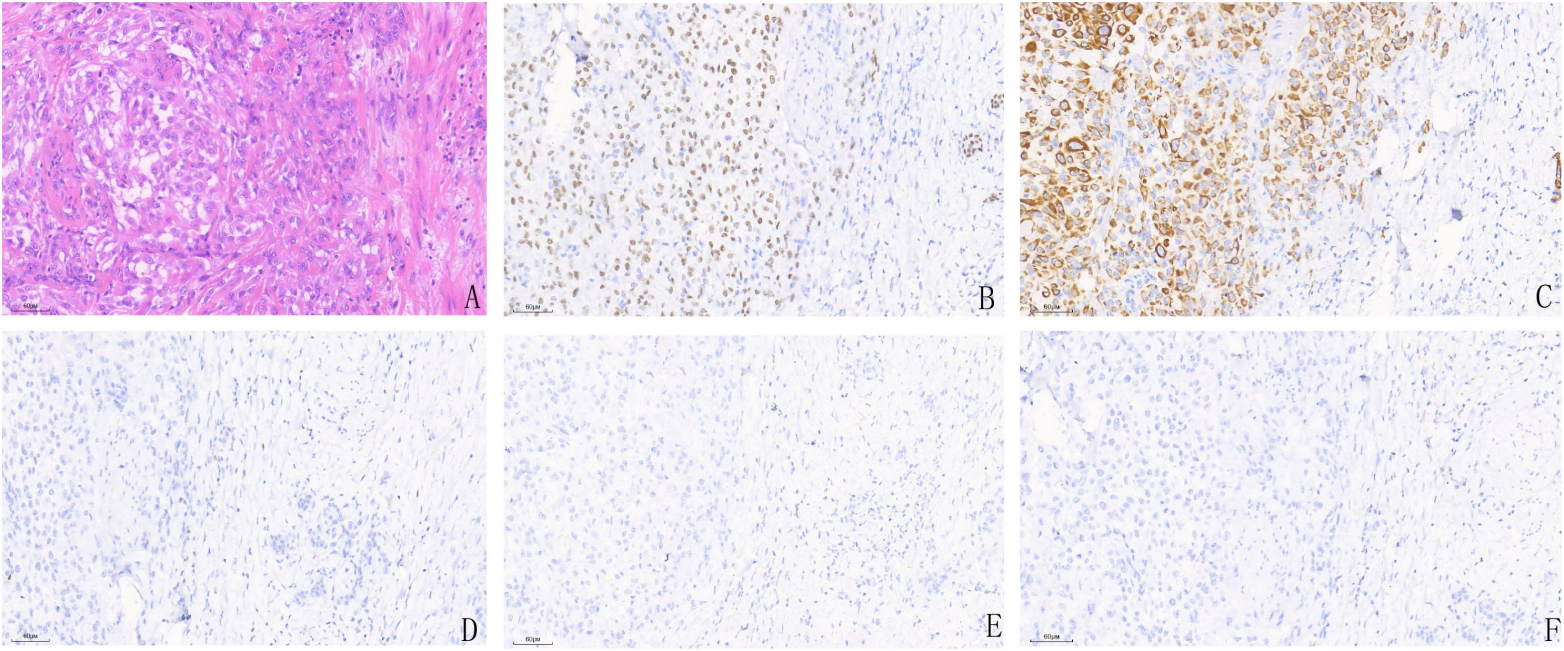
Histopathological findings of the bladder mass. **(A)** HE staining shows diffusely distributed and cord-like infiltrative tumor cells with frequent mitotic figures, consistent with invasive urothelial carcinoma. **(B–F)** Immunohistochemical staining reveals positive expression of P40 **(B)** and CK-pan **(C)**, while the tumor cells are negative for GATA3 **(D)**, CK20 **(E)**, and Uroplakin II **(F)**. CT: computed tomography; MPC, Multiple primary cancers; HE, Hematoxylinandeosin; CTX, cyclophosphamide; MALT, mucosa-associated lymphoid tissue; NCCN, National Comprehensive Cancer Network.

Histopathological examination with hematoxylin and eosin (H&E) staining revealed tumor cells arranged in diffuse sheets and cord-like invasive patterns, with frequent mitotic figures—characteristic features of invasive bladder carcinoma (**Figure 3A**). Immunohistochemical (IHC) analysis demonstrated that the tumor cells were positive for CK-pan, P40, and Ki-67 (approximately 5% positive). In contrast, the neoplastic cells were negative for GATA3, CK20, and Uroplakin II (**Figures 3B–F**). The final diagnoses were summarized as follows (1):High-grade invasive urothelial carcinoma of the bladder with squamous differentiation (pT2N0M0) (2); MALT lymphoma, stage IVA. The patient’s condition remained stable postoperatively with satisfactory recovery, and she was scheduled for regular follow-up visits within two months after discharge.However, she unfortunately died before the planned follow-up could be completed. Therefore, long-term follow-up data are unavailable.

## Discussion

The diagnosis of MPC is based on three classical criteria proposed by Warren and Gates (1, 2): each tumor must be histologically confirmed as malignant (2); all tumors must be independent, excluding the possibility of mutual metastasis; and (3) the tumors must arise at distinct anatomical sites. This definition underscores both the anatomical separation of the lesions and the exclusion of secondary metastases. MPC are categorized as *synchronous* when the interval between the diagnoses of two primary cancers is within six months, and as *metachronous* when the interval exceeds six months. The reported incidence of double or triple primary malignancies among MPC patients is approximately 0.5% (3). Moreover, compared with patients harboring a single primary cancer, those with MPC generally exhibit more aggressive disease behavior and poorer prognostic outcomes.

In the present case, we consider the bladder tumor fulfilled all Warren and Gates criteria for an independent second primary malignancy rather than metastatic disease. First, the histopathological features of the two tumors were entirely distinct, with the initial lesion representing pulmonary MALT lymphoma and the subsequent tumor diagnosed as high-grade invasive urothelial carcinoma with squamous differentiation. Second, no radiological or clinical evidence of systemic metastasis or direct extension was identified. Third, the interval between the diagnoses exceeded six years, supporting the classification as metachronous MPC.

From a pathological perspective,the diagnosis of urothelial carcinoma in this case was supported by both morphological and immunophenotypic findings.Immunohistochemical analysis of the resected bladder and tumor specimens revealed positivity for CK-pan and p40, while GATA3, uroplakin II, and CK20 were negative, with a Ki-67 proliferation index of approximately 5%. Histopathological examination demonstrated that the tumor was embedded within a dense fibrotic stroma and composed of irregular nests and cords of atypical squamoid cells, distributed in a prominent desmoplastic background. The tumor cells exhibited enlarged, hyperchromatic, and pleomorphic nuclei with variably prominent nucleoli and moderately eosinophilic cytoplasm. Intercellular bridges and focal keratinization were observed, although well-formed keratin pearls were absent. These morphological features are consistent with invasive squamous cell carcinoma with a marked stromal reaction and are also compatible with high-grade or squamous-differentiated variant urothelial carcinoma. Notably, prior studies have shown that GATA3 expression is substantially reduced in urothelial carcinoma with squamous differentiation, and that terminal differentiation–associated markers such as CK20 and uroplakin II may be decreased or lost in a subset of high-grade and/or invasive urothelial carcinomas (4, 5), which may partially explain the negative immunophenotype observed in the present case.

The pathogenesis of MPC remains incompletely understood. Several risk factors have been implicated, including genetic susceptibility, immune dysregulation and tumor immune evasion, accumulation of somatic mutations and aberrant gene expression, as well as prior exposure to radiotherapy, chemotherapy, or specific pharmacologic agents (6, 7). In addition, smoking, recurrent bladder infections, and chronic irritation are recognized as major contributors to bladder carcinogenesis.

The patient in this case received multiple chemotherapy regimens containing CTX following the diagnosis of MALT lymphoma. After metabolic activation by hepatic cytochrome P450 enzymes, CTX generates acrolein, which is excreted via the urinary tract. During bladder storage, acrolein interacts with urothelial DNA and cell membranes, inducing mutagenic alterations. Moreover, it triggers the release of pro-inflammatory mediators from injured epithelial cells, thereby recruiting macrophages and neutrophils and sustaining a chronic inflammatory microenvironment (8–10). Importantly, CTX exposure exhibits a well-documented dose-dependent association with an increased risk of bladder cancer (11, 12).

Notably, the cumulative CTX dose administered in this case was 9.6 g, which lies within the lower-dose spectrum. We hypothesize that the subsequent development of bladder cancer may not have been solely attributable to CTX exposure, but may also have been related to lymphoma-associated immune deficiency or immune dysregulation (13). Previous studies have indicated that MALT lymphoma survivors who received chemotherapy have a higher risk of developing second malignancies compared with those who did not receive chemotherapy (14).The resulting impairment of immune surveillance and disruption of immune homeostasis could diminish host defense capacity, facilitating malignant transformation (15, 16).Moreover, the immunosuppressed microenvironment may alter drug metabolism and clearance pathways, thereby prolonging systemic exposure and amplifying organ-specific toxicity (17, 18). Accordingly, we now present immune dysfunction as a plausible contributing factor rather than the primary driver.

Overall, these factors may act synergistically to predispose patients with MALT lymphoma to secondary malignancies, such as lung, colorectal, and bladder cancers (19). Nonetheless, further mechanistic studies are warranted to clarify the precise pathophysiological links underpinning this rare MPC phenotype.

Due to their non-specific clinical manifestations and typically insidious onset, MPC are frequently misinterpreted as metastatic disease (20). In its early course, MALT lymphoma usually lacks distinctive symptoms and may remain clinically silent for an extended period. Moreover, because MALT lymphoma can arise in diverse anatomical sites—such as the stomach, lungs, and thyroid—its initial presentations often mirror the functional disturbances of the involved organ. By contrast, early-stage bladder cancer most commonly presents as painless gross hematuria. A subset of patients may also experience irritative voiding symptoms, including urgency, frequency, and dysuria; however, these manifestations are similarly non-specific and can easily be mistaken for nephritis or urinary tract infections. The overlap of such subtle and heterogeneous symptoms highlights the diagnostic complexity of MPC and underscores the need for heightened clinical vigilance and comprehensive differential assessment.

The management of MPC requires close multidisciplinary collaboration among specialists in surgery, medical oncology, radiology, and pathology (21). Effective treatment planning should comprehensively account for tumor stage, anatomical location, and patient performance status. According to the National Comprehensive Cancer Network (NCCN) guidelines, recommended regimens for MALT lymphoma and other advanced low-grade B-cell lymphomas include single-agent rituximab, bendamustine plus rituximab, or combination protocols such as BR, CHOP, or FCR. In cases of relapsed or refractory disease, BTK inhibitors—such as orelabrutinib—may be incorporated into the therapeutic regimen (citation).For bladder cancer, surgical management remains the cornerstone of therapy, particularly for early-stage or locally advanced disease. The NCCN guidelines recommend transurethral resection of bladder tumor followed by postoperative intravesical chemotherapy for non–muscle-invasive bladder cancer (T1N0M0). In contrast, radical cystectomy is indicated for muscle-invasive disease, whereas neoadjuvant chemoradiotherapy may be considered in patients with locally advanced tumors (22, 23).

Previous studies have demonstrated that patients with MPC generally have a poorer prognosis than those diagnosed with a single malignancy (1). Prognostic outcomes are influenced by multiple variables, including the stage and biological behavior of each tumor, therapeutic response, and the patient’s overall performance status. In the present case, the patient received chemotherapy following the initial diagnosis of MALT lymphoma. Owing to multiple recurrences, her treatment regimen was subsequently modified several times. It was only after the onset of gross hematuria that a comprehensive urological evaluation was conducted, leading to the identification of the second primary malignancy.

Therefore, we underscore the importance of long-term follow-up in lymphoma patients to monitor both disease recurrence and the emergence of second primary malignancies. From a preventive standpoint, several clinical strategies may mitigate such risks. First, administration of alkylating agents should be accompanied by uroprotective compounds, such as mesna, to neutralize toxic metabolites. Second, high cumulative doses and concomitant use of multiple alkylating agents should be avoided whenever feasible, with preference given to less toxic alternatives, including targeted therapies (e.g., BTK inhibitors) or immunotherapies (e.g., PD-1 inhibitors) (24). In terms of surveillance, urological monitoring should be incorporated into the long-term management plan for lymphoma patients. High-risk individuals should undergo periodic urinary cytology, urine biomarker assays, and cystoscopy when clinically indicated, supplemented by imaging modalities such as ultrasonography or CT urography to facilitate early detection. A primary limitation of this study is its design as a single case report. Therefore, data collection from larger cohorts is warranted to establish standardized therapeutic protocols and enable more robust prognostic assessments.

In conclusion, this report presents a rare and instructive case of metachronous MPC involving MALT lymphoma and bladder cancer, providing valuable clinical insights that contribute to the existing literature on MPC. The underlying mechanisms responsible for the metachronous development of MALT lymphoma and bladder cancer remain incompletely elucidated and warrant further investigation to clarify the pathogenesis and develop effective management strategies.

## Data Availability

The original contributions presented in the study are included in the article/supplementary material. Further inquiries can be directed to the corresponding author.
